# Efficacy and Safety of 5-Aminolevulinic Acid Photodynamic Therapy for Treating Cervical and Vaginal Intraepithelial Neoplasia

**DOI:** 10.3390/pharmaceutics16050627

**Published:** 2024-05-07

**Authors:** Qin Han, Hongyan Guo, Zhangxin Wu, Jiaxin Shi, Xue Zhang

**Affiliations:** 1 Department of Obstetrics and Gynecology, Peking University Third Hospital, Beijing 100191, China; bysyghy@126.com (H.G.);; 2 National Clinical Research Center for Obstetrics and Gynecology, Peking University Third Hospital, Beijing 100191, China

**Keywords:** high-risk HPV, cervical intraepithelial neoplasia, vaginal intraepithelial neoplasia, photodynamic therapy, PDT, 5-aminolevulinic acid, ALA

## Abstract

Persistent HPV infections may cause cervical and vaginal intraepithelial neoplasia (CIN and VaIN). Traditional methods might destroy the structure and function of the cervix. 5-aminolevulinic acid photodynamic therapy (ALA-PDT) is a non-invasive targeted therapy. This study aims to evaluate the efficacy and safety of ALA-PDT for CIN and VaIN and the clearance of HPV. A retrospective study of 303 patients who confirmed CIN or VaIN and received ALA-PDT was conducted. All the patients were followed up at six and twelve months after treatment and then annually thereafter. The effect was evaluated through HPV genotyping, a cytology test, and colposcopy-directed biopsy if necessary. After ALA-PDT, the remission rates for CIN 2, CIN 3, VaIN 2, and VaIN 3 were 90.6%, 88.5%, 87.3%, and 77.8%. For CIN 1, the remission rate at the six-month follow-up was 93.1%. The total HPV clearance rates were 72.5% at the six-month follow-up and 85.7% at the 12-month follow-up. The most common adverse event was vaginal discharge. No severe adverse effect was observed. ALA-PDT is an effective and safe treatment for all grades of CIN and VaIN and is helpful in clearing HPV with minimal side effects. This treatment may not influence fertility and delivery.

## 1. Introduction

The incidence of high-risk HPV infection in women has increased annually all over the world, with a youth-oriented tendency, and it may cause intraepithelial lesions and cancer in the lower reproductive tract [1].

While organized screening programs have led to a decrease in cervical cancer incidence in many Western countries [2,3], China has experienced a significant increase in both the incidence and mortality rates of cervical cancer over the past few years [4].

Lower genital tract epithelial dysplasia includes low-grade squamous intraepithelial lesions (LSILs) and high-grade squamous intraepithelial lesions (HSILs). The former includes LSILs of the cervix and vagina (CIN1 and VaIN1), and the latter includes HSILs of the cervix and vagina (CIN2/3, VaIN2/3) [5]. Low-grade precancerous lesions are mostly associated with transient HPV infections and generally have a favorable prognosis. However, they require close follow-up due to the small risk that persistent LSILs may progress to higher-grade lesions (HSILs). HSILs, particularly CIN3 lesions, carry a higher risk of progressing to cervical cancer if left untreated and should therefore be actively managed [6]. The methods used include surgical resection and physical therapy. Cervical conization destroys the structure and may cause miscarriage and premature birth [7]. Vaginal lesions are widely distributed, and complete surgical removal is often difficult. Traditional physical therapies, such as freezing and CO_2_ laser treatment, have an efficacy rate of approximately 60–80% [8]. However, poor curative effects are found for patients with multiple lesions, wrinkled lesions, and lesions that cannot be flattened. The adjacent normal tissue of the lesion may also be damaged.

Photodynamic therapy (PDT) is a physical treatment technology which is based on the dynamic interaction of PS, a specific wavelength of light, and molecular oxygen, resulting in the selective destruction of the target tissue. The PDT treatment includes the administration of PS, which selectively accumulates in tumor tissue, followed by exposure to an appropriate wavelength light. Light-activated PSs react with oxygen and yield reactive oxygen species (ROS), such as superoxide anion radicals, O_2_^٠−^, hydroxyl radicals, ˙OH, and hydrogen peroxides, H_2_O_2_ (Type I reaction). PS can also transfer energy to triplet oxygen in the ground state (^3^O_2_) through a Type II reaction, to generate highly reactive singlet oxygen, ^1^O_2_. In the end, these cytotoxic photoproducts start a cascade of biochemical events, which can induce the damage and death of the target tissue. The extracellular tissue is not damaged.

PDT has been used as a new technology for treating tumors or precancerous tissues. Currently, in gynecological care, PDT can be used as a less invasive treatment for superficial lesions that are widespread or difficult to eradicate [9]. The first-generation PSs (mainly Photofrin) must be applied intravenously, and second-generation PSs, such as 5-aminolevulinic acid (ALA), are usually applied on the surface and may reduce systemic photosensitivity side effects [10]. Since the lower reproductive tract is easily exposed, it is feasible to use ALA-PDT for gynecological diseases such as vulva Lichen sclerosus and condyloma acuminata. However, this modality is not widely used for intraepithelial lesions [11].

In this study, the cases of patients who underwent photodynamic therapy for cervical or vaginal precancerous lesions in the gynecology clinic of the Third Hospital of Peking University from March 2020 to August 2023 were reviewed. The purpose of this investigation was to evaluate the efficacy and safety of PDT in treating cervical and vaginal intraepithelial lesions in the real world and to provide more references for its application.

## 2. Materials and Methods

### 2.1. Population

Between March 2020 and August 2023, patients who visited the gynecology clinic of Peking University Third Hospital and were diagnosed with cervical/vaginal intraepithelial neoplasia through colposcopy and pathological biopsy were selected as the study subjects. After discussion with the clinicians, patients who were treated with ALA-PDT were included in the study. Before treatment, all the patients signed an informed consent form for ALA-PDT treatment. The indications for treatment included cervical/vaginal high-grade squamous intraepithelial lesions (HSILs) confirmed by colposcopy and pathological biopsy (including cervical CIN2 or CIN3 and/or vaginal VaIN2, VaIN3) or persistent CIN1/VaIN1 lasting for more than one year with a strong willingness to be treated; colposcopy was adequate, and analyzable colposcopy images were retained; and endocervical curettage (ECC) did not suggest higher-grade lesions. The contraindications for treatment included coexistence or suspicion of cancer; porphyria or photosensitivity to red and blue light; severe medical comorbidities (such as abnormal liver and kidney function, coagulation abnormalities, heart failure, asthma attacks, etc.) that could not be controlled; and systemic acute infections or unstable conditions. During the treatment process, the clinical data of the patients, including their age, HPV infection type, ThinPrep cytology test (TCT), distribution characteristics of lesions, cervical transformation zone, vaginal microecology, fertility history, and surgical history, were collected.

### 2.2. HR-HPV Test, Cytological Examination, and Pathological Biopsy Guided with Colposcopy

We used a brush to collect the exfoliative epithelium cells of the vagina and cervix. The Cobas^®^ HPV test (Roche Molecular Diagnostics, Basel, Switzerland) was performed to detect 14 high-risk types of HPV DNA, including HPV 16, 18, 31, 33, 35, 39, 45, 51, 52, 56, 58, 59, 66, and 68. ThinPrep fixative solution was used to collect pap smear samples, and an automated stainer was applied. According to the latest cervical cancer screening strategies, women with abnormal HPV or cytology results were referred for colposcopy. Colposcopy was performed using a light-emitting diode (LED) colposcope (Jiangsu Xinrui Medical Technology Co., Ltd., Suzhou, China) with 20–40× magnification. The entire area of the abnormal epithelium was colposcopically inspected after the application of 3% acetic acid followed by iodine solution. Negative iodine test lesions were suspicious areas, and a biopsy was performed in the suspicious area for pathological examination. Colposcopy was performed after treatment.

### 2.3. ALA-PDT

First, the patients were placed in the lithotomy position. After vaginal cleansing with sterile 0.9% sodium chloride, an acetic acid test and iodine test were performed on the vagina and cervix under colposcopy to determine the scope of the lesions before treatment. Unprepared ALA dispersant (Shanghai Fudan Zhangjiang Bio-Pharmaceutical Co., Ltd., Shanghai, China) was stored in the dark. A 20% gel of ALA was prepared with a thermosensitive gel (Shanghai Tairan Bio-Pharmaceutical Co., Ltd., Shanghai, China) on an icebag, which made it possible to dissolve the ALA into the gel. One bottle of ALA (118 mg) was dissolved in 0.5 mL gel. As ALA is photosensitive, the gel must be made for immediate use. The single dose of ALA was 38 mg/cm^2^. Cotton gauze soaked with ALA gel was applied to the cervical surface, and a sterile sliver soaked with ALA gel was used to fill the endocervical canal to ensure that all the lesions were covered, especially if the lesion extended into the endocervical canal or the squamocolumnar junction of the cervix was invisible. Then, a condom filled with gauze was inserted into the vagina to keep the gauze and the sliver with the gel in place; 3.5 ± 0.5 h later, this was followed by 635 nm wavelength light treatment at a density of 80 mW/cm^2^ for 25–30 min with a focal distance of 5 cm, and an optical fiber was extended into the cervical canal. An LED photodynamic instrument (LED-IB, Wuhan Yage Optic and Electronic Technique Co., Ltd., Wuhan, China) was used. ALA-PDT was conducted every 7–14 days, avoiding the menstrual period. The therapeutic schedule was tailored to each patient’s condition, ranging from 1 to 3 courses of treatment. Each course consisted of three ALA-PDT sessions.

### 2.4. Evaluation of Therapeutic Efficacy and Follow-Up

For the patients with HSILs, colposcopy was performed 3–4 weeks after treatment. All the patients were followed up six and twelve months after treatment. The effect was evaluated through HPV genotyping, TCT, and colposcopy-directed biopsy if necessary, in accordance with the guidelines. The patient and doctor discussed whether more courses of PDT or other treatments would be required if the disease stabilized or progressed.

The primary endpoint was pathological regression immediately after, 6 months after, and 1 year after the completion of treatment. Women without evidence of SIL lesions following colposcopic examination or biopsy were considered to be in complete remission (CR). Disease remission referred to patients with repeated vaginal biopsy-confirmed LSIL. The patients with the same grade lesions were categorized as having persistent disease, while those with higher-grade histologic findings were categorized as having progressive disease. HR-HPV-related remission rates at 6 months after treatment were also assessed as the second endpoint. We also assessed the recurrence rate at the 6-month and 1-year follow-ups. Adverse symptoms were also recorded.

### 2.5. Statistical Analysis

We used uni- and multivariate regression analyses to assess factors affecting treatment outcomes and recurrence. All statistical analyses were performed using IBM SPSS Statistics version 24.0.

## 3. Results

### 3.1. Population Characteristics

The patients who received PDT to treat female genital tract intraepithelial neoplasia, including histologically confirmed CIN or VaIN, at the Third Hospital of Peking University Gynecology Outpatient Clinic from March 2020 to August 2023 were included in this study. The following data were extracted in addition to these demographic characteristics: HPV and TCT results, distribution of disease lesions, smoking history, and prior history of uterine surgery. To avoid confusion in the data analysis, no patients with concurrent CIN or VaIN were included. In total, 303 patients were included. The baseline data of patients are listed in Table 1.

### 3.2. Therapeutic Response

For HSILs (CIN 2/3), the patients received one course of PDT and underwent colposcopy 3–4 weeks later to evaluate the effect. If the disease remained or progressed, the patient and doctor discussed whether to apply more courses of PDT or other treatments. Table 2 shows the therapeutic response and categories for HSILs. According to the colposcopy results after treatment, the remission rates for patients with CIN 2 and CIN 3 were 90.6% (135/149) and 88.5% (23/26), respectively. For patients with LSILs (CIN 1), one course was recommended, and the remission rate at the six-month follow-up was 93.1% (61/72). Among these 26 CIN 3 patients, 7 had glandular involvement, 4 had 3 courses of PDT, and 3 had 2 courses of PDT; fortunately, all of them achieved disease remission.

For vaginal HSILs (VaIN 2/3), the patients received one course of PDT and underwent colposcopy 3–4 weeks later to evaluate the effect. If the disease remained or progressed, the patient and doctor discussed whether to apply more courses of PDT or other treatments. Table 3 shows the therapeutic response and treatment categories. According to the colposcopy results after treatment, the remission rates for the VaIN 2 and VaIN 3 patients were 87.3% (41/47) and 77.8% (7/9), respectively. For the patients with VaIN 2 or VaIN 3, the lesions located in the vaginal stump after hysterectomy seemed to be difficult to treat, although this difference was not statistically significant (*p* = 0.089).

### 3.3. Follow-Up Results and Recurrence Cases

After PDT treatment, 25 patients had persistent lesions and were treated with other methods. By the end of February 2024, all the patients reached the six-month follow-up point, but five patients were lost to the follow-up. The numbers of patients who reached the 1-year, 2-year, 3-year, and 4-year follow-up points were 61, 79, 57, and 42, respectively. Regarding HSILs, 12 cases recurred, and none of the patients with recurrence exhibited disease progression (Table 4).

### 3.4. HR-HPV Remission Rates

The total HPV clearance rate was 72.5% (216/298) at the 6-month follow-up and 85.7% (239/279) at the 12-month follow-up. Table 5 shows the remission rates of different HPV types at different follow-up time points. There was no significant difference among the groups (*p* > 0.05).

### 3.5. Adverse Effects

The adverse effects are summarized in Table 6. The most common side effect was increased vaginal discharge, which occurred in 62.38% (189/303) of patients. Other side effects included abdominal pain, vulvar pruritus, and vaginal bleeding. Abdominal pain was observed in 61.38% (186/303) of patients, while 50.49% of the patients complained of mild pain (VAS scores 1–4), and 9.57% complained of moderate pain (VAS scores 5–7). Furthermore, no patients experienced massive vaginal bleeding after treatment. All the adverse effects were tolerable, and no patients discontinued treatment because of adverse effects.

### 3.6. Pregnancy and Obstetric Outcomes

Notably, 27 patients successfully became pregnant, whether through assisted reproductive technology or naturally; 23 of these patients successfully delivered healthy fetuses (Table 7), and 4 patients were still pregnant at the time of submission. Notably, although pregnancy was excluded by a urine test before treatment, one CIN2 patient achieved pregnancy after one course of PDT. After sufficient communication, she decided to maintain her pregnancy. At the time of submission, she was 33 weeks pregnant, and her fetal examinations were normal.

## 4. Discussion

Cervical cancer has become the third leading cause of cancer death among young women [12]. Many studies have confirmed that persistent infection with high-risk human papillomavirus (hr-HPV) is a certain cause of intraepithelial lesions and cancer in the female lower reproductive tract [13]. Although the HPV vaccine has been developed, it is still difficult to cover the population in the short term. In 2012, the screening guidelines for the prevention and early detection of cervical cancer recommended the HPV test, which is more sensitive than cytology [14], as one of the primary screening methods [15]. According to the latest guidelines in China, cervical cancer screening should begin at age 25, with HPV testing alone or combined with cytology every five years, or cytology alone every three years [16].

With the changes in screening strategies, the identifications of high-risk HPV infection and intraepithelial lesions have increased annually all over the world, with a youth-oriented tendency [1]. Most HPV infections are transient and may resolve within two years. Some of these infections will persist over several years and lead to precancerous lesions [17]. Therefore, additional tests, such as the cytology test, HPV genotyping, and the p16/ki-67 dual-stained cytology test, are required to identify the HPV-positive women with progressive infections or precancerous lesions [18].

Cervical precancerous lesions should be treated both actively and properly. Forty to fifty percent of young patients (below 30 years old) with LSILs would regress; for women above 30 years old, 40% will have disease persistence for more than 1 year, and 10% or more will progress to HSILs within 1–2 years [19]. Continuous HPV infection, persistent LSILs, and repeated examinations may lead to extreme anxiety and the consumption of medical resources. Therefore, noninvasive therapies are ideal for eliminating persistent HPV infection, reversing persistent LSIL, reducing lesion progression, and alleviating patients’ anxiety. Surgical resection can destroy the integrity and function of the cervix in HSILs, increasing the incidence of cervical incompetence, abortion, and premature delivery during subsequent pregnancies. Traditional physical therapies, such as laser ablation, may also damage normal tissue, leading to complications, such as bleeding and contracture.

With the expanding coverage of cervical cancer screening and advancements in colposcopy techniques, there has been a significant increase in the detection rate of vaginal epithelial lesions. However, the actual incidence of VaIN may be underestimated due to the complex special anatomical structure and hidden nature of the lesions. The risk of VaIN 1 progressing to cancer is relatively low, while the risk of VaIN 2–3 progressing to invasive cancer is approximately 12% [20]. Furthermore, the risk of progression to invasive cancer can reach 16.7% in patients who have undergone hysterectomy [21]. Therefore, clinicians should pay more attention to the diagnosis and treatment of VaIN. Clinical treatments for VaIN 2–3 include laser cauterization, local electrocoagulation, and surgical resection.

These treatments for cervical and vaginal precancers may cause serious complications, such as severe vaginal stenosis, mucosal atrophy, and fibrosis. Thus, exploring minimally invasive treatment methods that maintain tissue structure integrity and have comparable results is highly important. This is especially true for patients who have fertility requirements, are concerned about invasive procedures, have recurrent disease after surgery, have difficulty undergoing secondary surgery, and have difficulty undergoing cone biopsy due to severe cervical atrophy after menopause.

Photodynamic therapy is a new minimally invasive technology. Research shows that it works through multiple mechanisms: (1) directly killing tumor cells, (2) damaging blood vessels, and (3) inducing antitumor immunity. As epithelial cells selectively absorb ALA, ALA mainly converts to protoporphyrin IX in the superficial cervical epithelium and is metabolized into cytotoxic reactive oxygen species under the excitation of light at specific wavelengths, causing apoptosis and necrosis. CIN is approximately confined to 2–3 mm of epithelial tissue above the basement membrane of the cervical epithelium. VaIN is confined to the vaginal epithelium at a depth of 0.1–1.5 mm. ALA can be absorbed by the entire epithelial layer, and 635 nm red light can penetrate the epithelium to a depth of 3–5 mm [22]. This makes it possible to treat CIN and VaIN with ALA-PDT.

Research has shown that the regression rate of ALA-PDT in treating LSIL is 84.88% [23]. Another study compared ALA-PDT with CO2 laser treatment for LSIL, and the results were similar at 4–6 months posttreatment. At 12 months posttreatment, however, the CR rate of the ALA-PDT group was significantly greater [24]. In our study, one course was recommended for LSILs, and the remission rate at the six-month follow-up was 93.1%, which is similar to the findings of other studies. Although there is a chance of spontaneous regression, the risks of disease persistence and progression increase with age [19]. ALA-PDT has broad application prospects in the treatment of LSILs. However, due to the possibility of the spontaneous regression of LSILs, clinicians should consider factors such as the duration of the lesion, HPV type, colposcopy findings, and personal preferences when selecting appropriate treatment options to avoid overtreatment.

With the promotion of PDT technology, its effectiveness in HSIL has been gradually verified. Relevant studies have reported that the efficacy rate of ALA-PDT for treating CIN 2 patients is 86.3–92% [25,26,27]. However, these studies were mostly small in terms of sample size and had relatively short follow-up periods and strict patient selection criteria; patients with multiple comorbidities, recurrence after resection, an extensive lesion area, immune dysfunction, and other characteristics were excluded. In contrast, our study included a large sample size and long follow-up time. The remission rate for patients with CIN 2 was 90.6%. Previous studies rarely included patients with CIN 3, whereas the present study included some cases after fully evaluating the condition and obtaining informed consent from the patients. The remission rate for patients with CIN 3 was 88.5%, which also supports the use of ALA-PDT in clinical treatment. As some patients had cervical canal lesions, a sterile sliver soaked with ALA gel and an optical fiber extending into the cervical canal were very important for covering all the lesions.

On the other hand, we should focus on identifying patients who are most likely to benefit from PDT treatment. Research has shown that cytology with HSIL/ASC-H, which indicates severe lesions, and lesion area are associated with the efficacy of ALA-PDT [28]. Therefore, a comprehensive evaluation of cytology and colposcopy should be conducted when selecting patients for ALA-PDT treatment. Additionally, some scholars believe that the extent of the lesion is related to its severity, and large lesions may not be excluded from microinvasive cancer. Patients with CIN 3 lesions that extend beyond two cervical quadrants visible under colposcopy are usually more inclined to undergo surgical treatment. The treatment effect should be closely monitored if ALA-PDT is chosen, and patients should be promptly informed of other treatment options if the response is poor.

For VaIN 2–3, our team has previously published articles on the effects and factors influencing ALA-PDT in the treatment of VaIN [29]. The results of this study are similar to those of previous studies. Factors such as the penetration and absorption rate of the drug, along with the adequacy of lesion exposure, are critical for effective treatment. Therefore, age and history of hysterectomy may affect the efficacy of ALA-PDT.

In addition, the efficacy and recurrence rates of PDT are closely related to the mobilization of the body’s immune system and are similar to those of traditional physical therapies. For patients with extended lesions, special locations, multifocal distribution, and recurrent episodes, the choice of treatment needs to be made particularly carefully. We also considered the possible reasons for failure. First, ALA may not be sufficiently applied to the surface of the lesion, resulting in some lesions not absorbing the photosensitizer. Second, the lesion site may be close to the cervical fornix or adhere to the vaginal wall, making it difficult for LED light to reach because light travels in a straight line. Finally, the depth of the intraepithelial lesion may be too deep for 635 nm of red light to penetrate. We also need to evaluate the lesions through colposcopy if the effect is not satisfactory. For patients with persistent HSILs after ALA-PDT, the treatment efficacy can be improved by increasing the treatment course if the visible residual lesion is significantly reduced.

The high-risk HPV virus integrates with the nuclear chromosomal genome of cervical and vaginal epithelial cells, leading to inactivation of the p53 gene and the subsequent loss of control over cell proliferation regulation, resulting in the development of precancer lesions and cancer [30]. Persistent HPV infection could be treated by drugs for the local area or by physical methods. Interferon inhibits HPV E6 and E7 gene expression but has several disadvantages, such as a low clearance rate and long-term administration [31]. HPV-infected cells can selectively absorb ALA, leading to protoporphyrin IX accumulation in infected cells. After light exposure, HPV is eliminated by reactive free oxygen, which selectively kills cells and prevents virus replication by oxygen-dependent cytotoxicity, causing chain breaks or base site disappearance in the viral nucleic acid [32].

An in vitro 2016 study revealed that PDT can induce apoptosis in human keratinocytes transfected with HPV16 E7 [33]. The current literature reports that the rate of HPV clearance after PDT in cervical precancerous lesions ranges from 53.4% to 94.4%. Cang et al. studied 57 patients with HPV infection treated with ALA-PDT for 6 months and reported impressive 100% clearance rates for HPV18 and 87.5% for HPV16. Additionally, the clearance rate for the other 12 high-risk HPV types was 48.8% [34]. In another report, 20 patients with high-risk HPV (HPV 16/18) infection received ALA-PDT treatment, resulting in 80% of patients achieving viral clearance. GuL and colleagues analyzed the HPV clearance rate in a population treated with PDT for LSIL. They reported that the rate of HPV remission was 64.34% at 3 months after PDT treatment and increased to 82.54% at 6 months posttreatment [23]. In our study, the total HPV clearance rate was 72.5% at the six-month follow-up and 85.7% at the 12-month follow-up. The clearance rate for the HPV 16 subtype is slightly higher than that for the HPV 18 subtype. With the extension of the follow-up time, the HPV clearance rate increases, and generally good results can be achieved after 6 months.

In addition, the female lower genital tract has its own microbial environment, known as vaginal microecology. It can be affected by HPV infection, leading to a decrease in local resistance and an increase in the HPV infection risk and pathogenesis, forming a vicious cycle. It has also been observed in clinical practice that most women with HPV infection have vaginitis. In photodynamic therapy, a red excitation light source is primarily used to activate photosensitizers, which then selectively destroy target cells through the generation of reactive oxygen species. As there is some emerging research into the potential anti-inflammatory effects of light therapy, it may possibly improve the vaginal microecology through red light irradiation, collaboratively eliminating the HPV virus. But further research is needed to explore the effects of photodynamic therapy on the vaginal microecology and its effectiveness against HPV.

In terms of adverse effects, abdominal pain, increased vaginal discharge, vulvar pruritus, and vaginal bleeding may occur, but all the patients tolerated these reactions, and no severe side effects occurred, indicating that PDT has high safety.

For patients with fertility requirements, whether treatment affects fertility and fetal and neonatal health needs to be considered. Traditional treatment methods have side effects such as cervical stenosis, cervical insufficiency, premature membrane rupture, and premature birth. According to research reports, the risks of premature birth after LEEP and CKC surgeries are 2% and 4%, respectively [35]. When the resection depth of surgery is greater than 10–12 mm, the risk of premature birth in patients increases threefold [7]. Although the incidence is low, successful pregnancy and delivery are the hopes of every woman with reproductive needs in the overall global context of declining fertility. Therefore, in addition to preserving the anatomical structure with appropriate treatment methods, it is equally important to consider whether medication and treatment affect subsequent fertility and fetal health.

One study analyzed 10 patients who received ALA-PDT treatment for cervical or vulvar lesions. None of the patients developed infertility, and all of the patients delivered healthy full-term infants [36]. Ahn TG et al. reported two cervical cancer patients who achieved full-term pregnancy and delivered after receiving ALA-PDT combined with other treatments [37]. Yang et al. treated five pregnant patients with genital warts using ALA-PDT, with a 100% efficacy rate. PDT had no adverse effects on the mothers or fetuses, and all the patients delivered healthy infants [38]. Our data show that ALA-PDT is a safe and effective treatment for female lower genital tract diseases without adverse effects on female fertility. Patients can successfully become pregnant and deliver healthy infants. In our study, one patient was found to be pregnant, and the results of her current fetal examinations were normal. Although there are no reports of fetal side effects caused by ALA during pregnancy, it is still necessary to determine whether the patients are pregnant and to keep them fully informed during treatment.

## 5. Conclusions

ALA-PDT is an effective and safe treatment for all grades of CIN and VaIN and is helpful for clearing HPV. This treatment may not affect subsequent pregnancy or delivery.

## Figures and Tables

**Table 1:** Baseline data of patients.

Lesion Grade	CIN 1 (N = 72)	CIN 2 (N = 149)	CIN 3 (N = 26)	VaIN 2 (N = 47)	VaIN 3 (N = 9)
**Age**	42 (35~56)	36 (25~53)	31 (25~33)	53 (37~67)	52 (41~56)
**Menopause**	5 (6.9%)	3 (2.0%)	0 (00.0%)	39 (82.9%)	8 (88.9%)
**Smoking or Passive Smoking**	32 (44.4%)	102 (68.5%)	12 (46.2%)	19 (40.4%)	2 (22.2%)
**History of Cervical Surgery**					
LEEP history	3 (4.2%)	32 (21.5%)	0 (00.0%)	1 (2.1%)	0 (21.5%)
CKC history	13 (18.1%)	27 (18.1%)	0 (00.0%)	2 (4.3%)	2 (22.2%)
Hysterectomy history	/	/	/	31 (65.9%)	7 (77.8%)
**HPV**					
HPV16 ± other high-risk HPV-positive	21 (29.2%)	53 (35.6%)	9 (34.6%)	12 (25.5%)	4 (44.4%)
HPV18 ± other high-risk HPV-positive	17 (23.6%)	37 (24.8%)	4 (15.4%)	10 (21.3%)	1 (11.1%)
HPV16 and 18 ± other high-risk HPV-positive	9 (12.5%)	19 (12.8%)	3 (11.5%)	6 (12.8%)	1 (11.1%)
Other high-risk HPV-positive	25 (34.7%)	29 (19.5%)	10 (38.5%)	18 (38.3%)	3 (33.3%)
Negative	0 (00.0%)	11 (7.4%)	0 (00.0%)	1 (2.1%)	0 (00.0%)
**TCT**					
NILM	44 (61.1%)	49 (32.9%)	3 (11.5%)	29 (61.7%)	2 (22.2%)
ASC-US/ASC-H	24 (33.3%)	47 (31.5%)	8 (30.8%)	8 (17.0%)	4 (33.5%)
LSIL	4 (5.6%)	53 (35.6%)	14 (53.9%)	10 (21.3%)	3 (33.3%)
HSIL	0 (00.0%)	0 (00.0%)	1 (3.8%)	0 (00.0%)	0 (00.0%)
**Pathological Characteristics**					
Single lesion of HSIL	/	68 (45.7%)	19 (73.1%)	42 (89.4%)	8 (88.9%)
Multiple lesions of HSIL	/	81 (54.4%)	7 (26.9%)	5 (10.6%)	1 (11.1%)
Glandular involvement	/	58 (38.9%)	6 (23.1%)	/	/

Abbreviations: LEEP (Loop Electrosurgical Excision Procedure), CKC (Cold Knife Conization), NILM (No Intraepithelial Lesion or Malignancy), ASC-US (Atypical Squamous Cells of Undetermined Significance), ASC-H (Atypical Squamous Cells, cannot exclude HSIL).

**Table 2:** Therapeutic response to HSILs.

Course	Total Number	Number/CIN	Therapeutic Response/Number	Number of CIN 2, Number of CIN 3
1	88	82 CIN 2	Complete remission 29	27 CIN 2, 2 CIN 3
		6 CIN 3	Disease remission 48	45 CIN 2, 3 CIN 3
			Persistent disease 11	10 CIN 2, 1 CIN 3
2	83	67 CIN 2	Complete remission 49	41 CIN 2, 8 CIN 3
		6 CIN 3	Disease remission 28	22 CIN 2, 6 CIN 3
			Persistent disease 6	4 CIN 2, 2 CIN 3
3	4	4 CIN 3	Disease remission 4	4 CIN 3

**Table 3:** Therapeutic response to VaINs.

Course	Total Number	Number/VaIN	Therapeutic Response/Number	Number of VaIN 2, Number of VaIN 3
1	34	32 VaIN 2	Complete remission 9	7 VaIN 2, 2 VaIN 3
		2 VaIN 3	Disease remission 21	21 VaIN 2
			Persistent disease 4	4 VaIN 2
2	21	15 VaIN 2	Complete remission 14	10 VaIN 2, 4 VaIN 3
		6 VaIN 3	Disease remission 3	3 VaIN 2
			Persistent disease 4	2 VaIN 2, 2 VaIN 3
3	1	1 VaIN 3	Disease remission 1	1 VaIN 3

**Table 4:** Characteristics of the 12 patients who experienced recurrence.

Patient No.	Original Disease Level	Courses of PDT	Time between Therapy and Recurrence (Months)	Recurrent Disease Level	Lesion Distribution	History
1	CIN 2	1	18	CIN 2	Single	NO
2	CIN 3	2	6	CIN 2	Single	NO
3	CIN 3	2	12	CIN 2	Single	NO
4	CIN 3	3	24	CIN 3	Multicentric	NO
5	CIN 3	2	12	CIN 2	Multicentric	NO
6	CIN 3	3	36	CIN 2	Single	NO
7	CIN 3	2	12	CIN 3	Multicentric	CKC
8	VaIN 2	1	12	VaIN 2	Multicentric	Hysterectomy due to CIN
9	VaIN 3	2	6	VaIN 2	Single	NO
10	VaIN 3	2	12	VaIN 2	Multicentric	Hysterectomy due to CIN
11	VaIN 2	2	48	VaIN 2	Multicentric	Hysterectomy due to CIN
12	VaIN 3	3	6	VaIN 2	Single	NO

**Table 5:** Remission rates of different HPV types at different follow-up time points.

HPV Types	6-Month Follow-Up	12-Month Follow-Up
HPV 16 infection	73.1%	73.8%
HPV 16 combined with 12 other high-risk HPV infections	82.7%	83.1%
HPV 18 infection	55.6%	57.9%
HPV 18 combined with 12 other high-risk HPV infections	76.0%	76.0%
12 other high-risk HPV infections	85.4%	89.7%
HPV 16/18 combined with 12 other high-risk HPV infections	57.1%	65.7%

**Table 6:** Description of the side effects.

Side Effect	Number of CINs (%)	Number of VaINs (%)
**Abdominal pain**	152 (61.54%)	34 (60.71%)
Mild (VAS 1–4)	130 (52.63%)	23 (41.07%)
Moderate (VAS 5–7)	21 (8.50%)	8 (14.29%)
Severe (VAS > 7)	1 (0.40%)	3 (5.36%)
**Vulvar pruritus**	11 (4.45%)	3 (5.36%)
**Increased vaginal discharge**	168 (68.02%)	21 (37.5%)
**Vaginal bleeding**	87 (35.22%)	9 (16.07%)
1–5 mL	87 (35.22%)	9 (16.07%)
Over 5 mL	0 (0%)	0 (0%)

Abbreviation: VAS (Visual Analogue Scale/Score).

**Table 7:** Pregnancies and labor in PDT-treated patients.

Patient No.	Disease Level	Courses of PDT	Time Difference between Therapy and Pregnancy (Months)	Method of Becoming Pregnant Naturally (NA) Intrauterine Insemination (IUI) In Vitro Fertilization–Embryo Transfer (IVF-ET)	Vaginal Delivery—VD or Caesarean Section—CC (Indications)	Child Development
1	CIN 1	3	3	IUI	VD	Normal
2	CIN 2	6	11	IVF-ET	VD	Normal
3	CIN 1	3	8	NA	VD	Normal
4	CIN 2	3	9	IUI	CC (labor stagnation)	Normal
5	CIN 2	6	15	NA	VD	Normal
6	CIN 2	3	2	IVF-ET	CC (twins)	Normal
7	CIN 3	6	7	IUI	VD	Normal
8	CIN 2	3	9	NA	VD	Normal
9	CIN 2	3	5	IUI	VD	Normal
10	CIN 1	3	4	IVF-ET	CC (twins)	Normal
11	CIN 1	3	6	NA	VD	Normal
12	CIN 2	3	2	IVF-ET	VD	Normal
13	CIN 2	6	3	IVF-ET	CC (labor stagnation)	Normal
14	CIN 3	6	7	IUI	VD	Normal
15	VaIN 2	3	16	NA	VD	Normal
16	CIN 1	3	13	NA	VD	Normal
17	CIN 2	3	8	IVF-ET	VD	Normal
18	VaIN 2	3	7	IUI	CC (fetal distress)	Normal
19	VaIN 2	6	9	NA	VD	Normal
20	VaIN 2	3	13	NA	VD	Normal
21	VaIN 3	6	7	IVF-ET	CC (fetal macrosomia)	Normal
22	CIN 2	3	8	NA	CC (complication)	Normal
23	CIN 3	6	3	IVF-ET	VD	Normal

## Data Availability

Data are unavailable due to privacy.
